# Usual Protein Intake Amount and Sources of Nursing Home Residents with (Risk of) Malnutrition and Effects of an Individualized Nutritional Intervention: An *enable* Study

**DOI:** 10.3390/nu13072168

**Published:** 2021-06-24

**Authors:** Johanna Seemer, Dorothee Volkert, Daniela Fleckenstein-Sußmann, Stephanie Bader-Mittermaier, Cornel Christian Sieber, Eva Kiesswetter

**Affiliations:** 1 Institute for Biomedicine of Aging, Friedrich-Alexander-Universität Erlangen-Nürnberg, 90408 Nuremberg, Germany; dorothee.volkert@fau.de (D.V.); cornel.sieber@fau.de (C.C.S.); eva.kiesswetter@fau.de (E.K.); 2 Fraunhofer Institute for Process Engineering and Packaging, 85354 Freising, Germany; daniela.fleckenstein@ivv.fraunhofer.de (D.F.-S.); stephanie.mittermaier@ivv.fraunhofer.de (S.B.-M.); 3 Department of Medicine, Kantonsspital Winterthur, 8401 Winterthur, Switzerland

**Keywords:** malnutrition, nursing home, protein intake, food source, individualized intervention

## Abstract

Nursing home (NH) residents with (risk of) malnutrition are at particular risk of low protein intake (PI). The aim of the present analysis was (1) to characterize usual PI (total amount/day (d) and meal, sources/d and meal) of NH residents with (risk of) malnutrition and (2) to evaluate the effects of an individualized nutritional intervention on usual PI. Forty residents (75% female, 85 ± 8 years) with (risk of) malnutrition and inadequate dietary intake received 6 weeks of usual care followed by 6 weeks of intervention. During the intervention phase, an additional 29 ± 11 g/d from a protein-energy drink and/or 2 protein creams were offered to compensate for individual energy and/or protein deficiencies. PI was assessed with two 3-day-weighing records in each phase and assigned to 4 meals and 12 sources. During the usual care phase, mean PI was 41 ± 10 g/d. Lunch and dinner contributed 31 ± 11% and 32 ± 9% to daily intake, respectively. Dairy products (median 9 (interquartile range 6–14) g/d), starchy foods (7 (5–10) g/d) and meat/meat products (6 (3–9) g/d) were the main protein sources in usual PI. During the intervention phase, an additional 18 ± 10 g/d were consumed. Daily PI from usual sources did not differ between usual care and intervention phase (41 ± 10 g/d vs. 42 ± 11 g/d, *p* = 0.434). In conclusion, daily and per meal PI were very low in NH residents with (risk of) malnutrition, highlighting the importance of adequate intervention strategies. An individualized intervention successfully increased PI without affecting protein intake from usual sources.

## 1. Introduction

In many older adults, protein intake is below recommended amounts [1], but adequate intake is crucial for maintaining muscle mass and function [2,3]. Additionally, in older age, protein requirements appear to be higher due to a high prevalence of inflammation, wounds, infections, critical illness [4,5,6] and reduced anabolic response after protein ingestion [7,8]. It is evident that older adults require more protein compared to younger individuals to maximally stimulate postprandial muscle protein synthesis [7].

To improve protein intake in older adults, various dietary interventions are available, e.g., enrichment of meals and oral nutritional supplements [9,10,11]. Currently, the ideal quality, quantity and timing of protein interventions for the maintenance or growth of skeletal muscle are discussed [12,13]. In terms of quality, animal protein is more effective than plant protein in inducing anabolic responses [12,14,15]. The amount of protein intake for healthy adults aged 65 and older suggested by nutrition societies [16,17] and expert groups [4,5] ranges between 0.8–1.2 g per kg body weight (BW) per day (d). Regarding timing and the amount of protein at each meal, Bauer and colleagues [4], based on Paddon-Jones and Rasmussen [18], have recommended that older people should consume at least 25 g of protein per meal to stimulate postprandial muscle protein synthesis.

It is relevant to consider these three aspects regarding usual protein intake to provide adequate nutritional support. Corresponding previous literature has mainly focused on community-dwelling older adults [1,4,19,20], and data for nursing home residents, a group at particular risk of low protein intake [21], is limited [21,22,23]. In a Dutch study, daily protein intake of institutionalized persons (58 g/d, age 80 years) was about 20% lower compared to community-dwelling (71 g/d, age 77 years) or frail (71 g/d, age 79 years) older adults, with milk and dairy products being the main source at breakfast, lunch and snacks [21]. The highest protein intake was observed at dinner (24 ± 12 g/d) [21]. In a Spanish study with institutionalized older adults (mean protein intake 83 g/d, age 83 years), poultry and milk were the main animal-based and bread the main plant-based protein sources [23]. Protein intake of German nursing home residents was about 49 g (age 86 years) in women and 59 g (age 81 years) in men [24].

Nutritional support should thus aim at optimizing dietary intake without negatively affecting the consumption of usual foods, as a reduced intake of regular food components might attenuate potential beneficial effects, and in daily routine intake from usual sources is preferred over intervention products [25]. When reporting effects of dietary intervention concepts for nursing home residents, it is often not specified whether or how these interventions influence protein intake from usual food sources. In hospitalized older adults [26], patients after discharge [25], and nursing home residents without nutritional risk [27], intake of dietary supplements high in protein did not change usual protein intake. However, in an earlier study with nursing home residents at nutritional risk, habitual protein intake was negatively affected by an enrichment with multi-nutrient oral nutritional supplements [28].

Thus, the aim of the present analysis was (1) to characterize usual dietary protein intake of German nursing home residents with (risk of) malnutrition and low dietary intake regarding total amount per day and per meal as well as amount from sources per day and per meal and (2) to evaluate the effects of an individualized protein-energy intervention on these aspects.

## 2. Materials and Methods

### 2.1. Study Design and Participants

The present analysis is based on data from a single-arm intervention study conducted in two nursing homes in Germany (Nürnberg), who belonged to the same provider and received the meals from one central kitchen. We used a sequential design in which each participant served as his/her own control. Residents received six weeks of usual nutritional care followed by six weeks of individualized nutritional intervention.

The study was registered at drks.de (Reference: DRKS00017584) and conducted in accordance with the Declaration of Helsinki about ethical principles for medical research. Approval was given by the ethics committee of the Friedrich-Alexander Universität Erlangen-Nürnberg (Reference: 71_19 B). All participants or their legal representatives provided written informed consent.

We screened all 306 residents permanently living in the participating nursing homes. Inclusion criteria were malnutrition or risk of malnutrition. Malnutrition was defined according to the Mini-Nutritional Assessment Short-Form (MNA-SF) with a score ≤ 7 points [29]. Risk of malnutrition was defined either by MNA-SF 8–11 points and a reduced score in at least one of the nutrition-related MNA-SF items (decreased food intake, unintentional weight loss, psychological stress, or acute disease, low Body Mass Index (BMI)) or by receiving texture-modified meals and a reduced score in one of the described MNA-SF items. Exclusion criteria were age <65 years, enteral or parenteral nutrition, acute illness, terminal stage of life and BMI ≥30 kg/m^2^.

Fifty-five residents were included, and 50 completed the study (4 deceased and 1 had a hospital stay > 1 week during the intervention phase). For this analysis, residents without calculated energy and/or protein deficiency were excluded, resulting in 40 participants, 13 from one and 27 from the other nursing home (For a flow chart of the recruitment process see [30]).

### 2.2. Participant Characteristics

Research associates extracted sex, age, number of medications, chronic diseases, body height and type of meals (regular or texture-modified) from care records.

Body weight was measured in daily routine by nursing staff to the nearest 0.1 kg with residents wearing regular indoor clothing using available chair or lift scales.

Clinical Frailty Scale (CFS, 1–9, very fit to terminally ill [31]), activities of daily living (Barthel-Index, 0–100, total to no dependency [32]), dementia (severe, mild, or no dementia) and mobility (bed/ chair bound, able to get out of bed/chair but does not go out, or goes out) were assessed in interviews with the nursing staff.

### 2.3. Usual Care

The central kitchen of the nursing homes offered three main meals (breakfast, lunch, dinner) and additional snacks.

Breakfast and dinner were based on bread and pastries with butter, sausage, cheese and jam (only for breakfast), with an optional yoghurt. Residents with chewing and/or swallowing difficulties received porridge. For breakfast, nursing staff prepared the porridge based on milk and instant cereal or fruit flakes. For dinner, the kitchen produced the porridge based on milk and either oats, maize, semolina, millet or rice, and served it with fruit compote. All residents could order an additional soup or daily specials (e.g., raw vegetable salads, pickled salads or fish) for dinner.

For lunch, residents chose their meals weekly out of an eight-week plan with three menu lines (one vegetarian) with an additional dessert (e.g., fruit curd, pudding). Water and juice were available at all times. Snacks throughout the day were a choice of fruit or yoghurt and an afternoon snack with varying baked goods (e.g., plane cake, milk snack, apple pie). Additionally, residents could consume meals and snacks brought by family and friends.

### 2.4. Individualized Intervention

During the intervention phase, we offered three intervention modules, a protein-energy drink, a sweet and a savory protein cream, in addition to usual nutritional care. Details of the intervention concept are described elsewhere [30].

The protein-energy drink was provided in a 250 mL ready-to-drink preparation containing 220 kcal and 22 g protein (with 20 g whey protein). Nursing staff offered the protein-energy drink according to participants’ liking at once (at breakfast, as morning or afternoon snack) or in several portions spread throughout the day.

The protein creams were offered in a sweet and a savory variant, each portion of 40 g containing 125 kcal and 10 g of whey protein, on the lunch tray of the respective participant. Nursing staff stirred the savory protein cream into the soup or main dish. The sweet protein was consumed either in pure form, with the dessert at lunch or with the cake as an afternoon snack.

Intervention modules were offered single or combined in four levels to compensate for individual energy and/or protein deficiencies (Supplement Table S1) [30]. Deficiencies were calculated as the difference between intake and requirements [16,33]. In the lowest level, only the sweet protein cream, and in the highest level, all modules were provided daily. The intervention was mainly offered at breakfast (protein-energy drink) and lunch (all modules, max. +42 g protein and +470 kcal).

### 2.5. Dietary Intake Assessment

Dietary intake was assessed using 3-day weighing records, during the first and the last week of the usual care and the intervention phase, respectively. Each component of every meal and all leftovers were weighed with digital kitchen scales (Soehnle 67080 Page Profi) to the nearest 1 g by trained nutritional scientists. When weighing of food (e.g., snacks brought by family or friends) or leftovers (e.g., due to mixing of components on the plate) was not possible, amounts were estimated by household measures. All energy-containing drinks were documented. Snacks during the night were registered by nurses using household measures. All snacks consumed during the day were combined as snack moments for the analysis.

Energy [kcal] and protein [g] intake from every food item was calculated with EbisPro 2016 (Willstätt-Legelshurst, Germany, German Nutrient Data Base Version 3.02) based on the consumed amount. One nutritionist conducted the data entry, which was verified by another.

### 2.6. Protein Intake

Protein intake was categorized into the following 12 protein sources [19]:

- Six plant-based protein sources: (1) starchy foods (e.g., rice, potatoes, noodles), (2) fruit and vegetables, (3) pulses (incl. stew with lentils or peas), (4) pastry, confectionery mainly vegetable (e.g., croissant, apple pie), (5) nuts and seeds and (6) other mainly plant-based protein sources (e.g., dumplings, beer) and
- Six animal-based protein sources: (7) meat and meat products, (8) dairy and dairy products, (9) eggs and egg products, (10) pastry, confectionery mainly animal (e.g., porridge, cream pies, pudding), (11) fish and seafood and (12) other mainly animal-based protein sources (e.g., stew with meat)

For mixed foods, the categorization was determined by the component delivering the greatest amount of protein. All protein intake results are based on individual values from six days of assessment from each phase. Two 3-day weighing records within each phase were combined as dietary intake did not differ. Only protein sources that contribute at least 5% to daily or meal protein intake are reported numerically.

### 2.7. Data Analysis and Statistics

Statistical analysis was performed with SPSS Version 26 (IBM SPSS Statistics, Chicago, IL, USA). Participants’ characteristics (continuous variables) and protein intake data were tested for normality using Q-Q-Plots and Kolmogorov-Smirnoff-Test. Participants’ characteristics are presented as *n* (%), mean and standard deviation (SD) (normally distributed) or median and interquartile range (IQR) (non-normally distributed).

Protein intake is presented as mean (SD) and median (IQR). Differences between usual care and the intervention phase in total protein amount (normally distributed) were tested using *t*-test for paired samples and in amount from protein sources (non-normally distributed) by Wilcoxon-signed rank test. The total amount of protein intake is presented in g/d, g/kg BW/d and g/meal. The amount from protein sources is described as g/d and g/meal. The contribution (%) of food sources to daily and mealtime intake is described as mean (SD). The number of participants consuming more than 1 g/kg BW/d and more than 25 g in at least one meal per day in the usual care and the intervention phase are described as *n* (%).

## 3. Results

### 3.1. Study Population

The participants had a mean age of 85 years, 75.0% were female and 85.0% were mildly or severely demented, and 27.5% were malnourished and 72.5% at risk of malnutrition (Table 1). Mean energy intake was 1404 kcal/d. One in three participants received texture-modified meals.

### 3.2. Daily and Mealtime Protein Intake Amount

#### 3.2.1. Usual Care Phase

**Figure 1:**
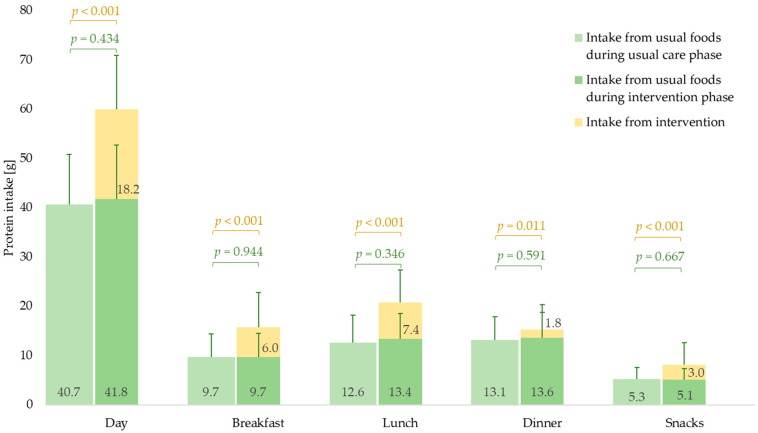
Mean daily and mealtime protein intake (g) of nursing home residents with (risk of) malnutrition during usual care (left bars) and intervention phase (right bars) from usual food sources (green) and intervention products (yellow) (*n* = 40). Protein intake from usual food sources refers to consumed usual foods averagely consumed during six days of assessment in each phase. *t*-test for paired samples. Positive standard deviations are shown for the protein amount from usual food sources and for the amount from usual food sources plus intervention.

During the usual care phase, mean protein intake was 40.7 ± 10.1 g/d and 0.70 ± 0.18 g/kg BW/d (Figure 1, Supplement Table S2).

Intake was highest at dinner (13.1 ± 4.7 g) and lunch (12.6 ± 5.6 g). Protein intake at dinner contributed 32 ± 9% and at lunch 31 ± 11% to daily intake. The lowest intake was during snack moments, which contributed 13 ± 5% to daily protein intake.

Only one participant (3.0%) consumed more than 1.0 g/kg BW/d, and four (10.0%) reached the threshold of 25 g at one meal, two of them at lunch, one at breakfast, and one at dinner.

#### 3.2.2. Intervention Phase

During the intervention phase, 29.0 ± 10.7 g protein (and 322 ± 116 kcal) per day were offered, of which 18.2 ± 9.6 g/d were consumed (Figure 1, Supplement Table S2). During the intervention phase, protein (59.4 ± 10.9 g/d vs. 40.7 ± 10.1 g/d, see Figure 1) and energy intake (1621 ± 288 kcal/d vs. 1404 ± 327 kcal/d, *p* < 0.001) were significantly higher compared to the usual care phase. Mean protein intake from the protein-energy drink was 10.8 ± 9.2 g/d, from the sweet protein cream 5.1 ± 3.4 g/d and from the savory protein cream 2.2 ± 2.8 g/d (Table 2).

In accordance with our intervention concept, the highest additional intake was at lunch and breakfast (Figure 1, Supplement Table S2). At all meals during the intervention phase, the mean total protein intake (incl. intervention) was significantly higher than during the usual care phase (Figure 1).

Twenty-five (62.5%) participants consumed more than 1.0 g/kg BW/d. Fourteen residents (35.0%) had a protein intake of 25 g or higher in at least one meal, including ten at lunch and two at breakfast.

Total daily and mealtime protein intake from usual food sources did not change when NH residents received the individualized intervention (Figure 1, Supplement Table S2).

### 3.3. Daily and Mealtime Protein Intake by Sources

#### 3.3.1. Usual Care Phase

**Figure 2:**
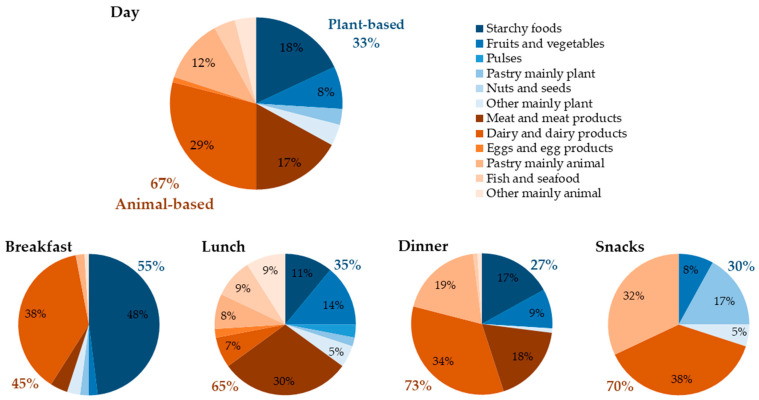
Mean contribution of 12 protein sources to daily and mealtime protein intake of nursing home residents with (risk of) malnutrition during the usual care phase (*n* = 40) based on individual means of two 3-day weighing records. Only usual food sources that contributed at least 5% to daily or mealtime intake are presented numerically. Blue = Plant-based protein sources, Red = Animal-based protein sources.

Residents consumed about two thirds of their daily and mealtime protein intake from animal- and about one third from plant-based sources, except for breakfast where about half was derived from plant-based sources (Figure 2). In total, five food groups contributed 84% to daily protein intake, of which dairy products (29 ± 14%), followed by starchy foods (18 ± 7%) and meat and meat products (17 ± 11%) were the main sources (Figure 2, Table 2).

At breakfast, starchy foods and dairy products accounted for 86% of protein intake (Figure 2, Table 2). At lunch, the diversity of sources was highest (eight food groups contributed more than 5% to meal intake). Meat and meat products accounted for almost one third (30 ± 19%) of protein intake (Figure 2). At dinner and snack moments, dairy products (34 ± 18% and 38 ± 21%) and pastry, confectionery mainly animal (19 ± 26% and 32 ± 21%) contributed the highest share (Figure 2). Dairy products were the main protein source at all mealtimes (up to 38 ± 25%), except lunch (7 ± 12%).

#### 3.3.2. Intervention Phase

Daily intake from usual protein sources did not change during the intervention phase (Table 2). At meal level, for some protein sources small, but significant changes were found: Protein intake of dairy products increased at lunch and decreased at dinner. At snack moments, protein consumption from pastry, confectionery mainly animal was higher and intake from fruit and vegetables was lower in the intervention compared to the usual care phase (Table 2).

## 4. Discussion

The present analysis showed that protein intake was very low among nursing home residents with malnutrition or risk of malnutrition and inadequate dietary intake. Only one participant consumed ≥ 1.0 g/kg BW/d during the usual care phase. Lunch and dinner were the meals with the highest protein intake, and the main usual protein sources were dairy products, meat and meat products, and starchy foods. The individualized nutritional intervention, primarily offered at breakfast and lunch, successfully improved protein intake without compromising the intake of usual food sources.

Compared to studies in Dutch and Australian institutionalized older adults with unknown nutritional status protein intake was about 40% higher than in our sample (56–60 g/d vs. 41 g/d) [21,22]. Regarding age, gender, cognitive, and functional status, our population was comparable to other nursing home populations at nutritional risk [34,35], but protein intake was lower, explained by the fact that we only included residents with calculated energy and/or protein deficiency. To adequately improve protein intake, nutritional interventions considering individual requirements are needed [6].

In the present study, 29 ± 11 g protein were additionally offered per day, which is within the range of other nutritional intervention studies in nursing home residents (9–45 g protein/d [34,35,36,37]). Overall compliance with the intervention products was acceptable (median intake between 44–96%), as reported previously [30]. The intervention was able to increase the proportion of participants consuming more than 1.0 g/kg BW/d (63% vs. 3%) and those reaching the suggested mealtime threshold of 25 g in at least one meal per day (35% vs. 10%), although the latter was no specific objective of the study. It is suggested that older adults should consume at least one high-protein meal per day to support maintenance or growth of skeletal muscle mass and strength [13]. Different mealtime thresholds, ranging from 20–45 g [4,18,38,39,40,41] or 0.25–0.40 g/kg BW [7,42,43,44], are described in the literature and analyzed in observational studies with regard to physical function and muscle mass. However, clear results on how much protein per meal is needed to maintain muscle mass and strength in older adults are still lacking. Especially for nursing home residents, there is a need to further study the influence of the amount of protein intake at each meal on physical parameters in the long term.

In the context of adequate timing of interventions, the satiating effect of protein is worth mentioning [28,45], as reduced intake of usual food components should be avoided. International guidelines recommend offering supplements between the meals and following individual taste and eating capacities [6]. Our intervention was offered with meals as well as between mealtimes and attempted to address individual dietary patterns (i.e., usually ingested amount of food at mealtimes and taste preferences) [30]. It effectively increased daily and mealtime protein intake without compromising the intake of regular food components. For other intervention concepts that were also successful in increasing protein intake in nursing home residents, it is unclear if or how the intake of the usual dietary components was influenced, as this aspect is usually not described [34,36,37,46].

The decision to offer supplements primarily at breakfast and lunch was based on our objective to integrate the intervention into the existing catering concept, where especially for dinner preparation the availability of kitchen and nursing staff was limited. It should be noted that offering supplementation at dinner could have additionally improved protein intake. However, offering an intervention at breakfast and lunch was supported by previous findings in community-dwelling older adults, where this was shown to improve daily protein intake [13,20,47,48,49]. In our population as well as in other institutionalized [21,50], community-dwelling [19,20,51], and rehabilitating [52] older adults, lunch or dinner were the mealtimes with the highest and breakfast with the lowest protein intake. Thus, offering supplements at breakfast may allow more evenly distributed protein intake throughout the day. Research in institutionalized and community-dwelling older adults showed that individuals consuming protein evenly distributed over the day tended to have a higher protein intake [22] and were less frail [53].

Regarding protein sources, it should be noted that we based the selection of food groups on data from community-dwelling older adults as we assumed comparability of protein sources and intake distribution throughout the day, an assumption that was confirmed. Dairy products, meat and meat products (incl. poultry) and starchy foods (incl. bread and cereal products) were the main sources of daily protein intake in our sample as well as in other institutionalized and community-dwelling older adults without particular nutritional risk [19,20,21]. Among Dutch community-dwelling older adults with low (<0.8 g/kg BW/d) protein intake, meat contributed less to total daily intake when compared to individuals with high (≥0.8 g/kg BW/d) intake [20]. Consistent with this finding, meat contributed less to daily intake in our participants at nutritional risk compared to another sample of institutionalized older adults with unknown nutritional risk (17% vs. 29%) [21].

The main sources at breakfast in the present and the previously mentioned studies of institutionalized and community-dwelling older adults were starchy foods and dairy products [19,20,21]. The greatest variety in protein sources was at lunch, with meat and meat products being the main source, in our nursing home sample as well as in community-dwelling older adults [19]. At dinner, our participants consumed most protein from dairy and dairy products (34%), followed by meat and meat products (18%), whereas in the other studies, meat ranked first among protein sources [19,20,21]. Snacks in our sample, as in other institutionalized [21] and community-dwelling older adults [19,20], were mainly dairy products and pastry. Overall, the differences in protein sources between studies and settings can be considered marginal, especially when taking individual, regional and country-specific dietary habits into account [1,54].

In total, the intervention did not affect daily protein intake from usual sources, and changes in the food intake at meals were small. Observed differences between usual care and intervention phase might be related to variations in the provided meals, as protein sources and distribution of protein intake throughout the day are largely dependent on the nursing home’s catering concept.

In the present sample (67%), Dutch institutionalized (71%) [21] and German community-dwelling (61%) [19] older adults without particular nutritional risk, the main protein sources were animal-based. This is relevant regarding quality, as animal protein is more favorable in inducing postprandial muscle protein synthesis compared to plant protein [12,14,15]. Moreover, at all meals, except lunch, the main protein source was dairy, and thus whey protein, which is particularly effective in contributing to muscle protein synthesis due to its high content of essential amino acids such as leucine [15,55,56]. For this reason, we have based our intervention modules on whey protein. Particularly for individuals with low protein intake, quality is important as consumption of low-quality protein may require higher amounts to prevent loss of muscle mass and strength [57].

Diets high in animal products are however associated with, e.g., the onset of chronic, especially cardiovascular, diseases [58]. Diets high in plant protein are generally associated with positive health effects [59,60,61]. Furthermore, plant-based diets appear to have a reduced environmental footprint and are therefore more sustainable, an aspect becoming more and more important [15,62]. To take these aspects into account and still provide older adults with high-quality protein, further research needs to be conducted on the effects of combining different plant protein sources, combining plant and animal sources or plant sources with essential amino acids [63,64].The present analysis has several limitations that are important to mention. First, we describe a small and specific group of participants, which limits generalizability. However, as research in nursing homes is difficult [65] and thus information is sparse, our high-quality intake data using weighing records significantly improved the level of knowledge on this topic. Second, although we were able to increase the protein intake per day and per meal, and the number of residents reaching suggested daily and mealtime amounts, intake was still insufficient in many participants. To further increase protein intake, it might have been necessary to offer more protein to residents with very low intake or to additionally offer supplements at dinner. Third, we did not perform blinding or randomization, which may have influenced the results. Blinding of the nursing staff was not possible, as they carried out the nutritional intervention, which could not be replaced by placebo products. Furthermore, we did not randomize participants into an intervention and a control group due to the expected high heterogeneity at resident and ward level and for ethical reasons: In our sequential design, it was possible that each resident received one intervention.

## 5. Conclusions

Our results highlight the importance of improving protein intake for the high-risk group of nursing home residents with (risk of) malnutrition and low dietary intake, as intake was particularly low in our German population. Differences in protein intake between our participants and older adults without nutritional risk mainly refer to the total amount of protein and the lower intake of meat, but not to other sources or the distribution throughout the day. Our intervention with high-quality protein, mainly offered at breakfast and lunch, successfully improved daily and mealtime protein intake without affecting regular dietary intake. Future studies aiming to optimize dietary protein intake in nursing home residents should consider the timing, quantity, and quality of usual protein intake and investigate the effect on functional and clinical parameters.

## Figures and Tables

**Table 1:** Participants’ characteristics at baseline.

*n* = 40		*n* (%)	Mean ± SD/Median (IQR)
**Age** (years)			85.2 ± 7.8
**Sex**, female		30 (75.0)	
**Number of medications per day**			6.0 (4.0–9.5)
**Barthel-Index** (points)			35.0 (7.5–60.0)
**CFS** (points)			6.9 ± 0.7
**Dementia**	Severe	22 (55.0)	
	Mild	12 (30.0)	
	No	6 (15.0)	
**Mobility**	Bed or chair bound	15 (37.5)	
	Able to get out of bed/chair	17 (42.5)	
	Goes out	8 (20.0)	
**Body weight** (kg)			59.3 ± 10.5
**BMI** (kg/m^2^)			22.0 (19.7–23.9)
**Energy intake** (kcal/d)			1404.3 ± 327.1
**Texture-modified meals**, yes		13 (32.5)	
**MNA-SF**	Malnourished	11 (27.5)	
	Risk of malnutrition	29 (72.5)	

IQR = Interquartile range, BMI = Body mass index, CFS = Clinical Frailty Scale (1–9, very fit to terminally ill), MNA-SF = Mini-Nutritional-Assessment Short-Form (Malnourished 0–7 points, Risk of malnutrition 8–11 points), SD = Standard deviation.

**Table 2:** Protein amount (g) by protein sources per day and per meal of nursing home residents with (risk of) malnutrition during usual care and intervention phase (*n* = 40).

		Usual Care Phase				Intervention Phase				
		Mean	SD	Median	IQR	Mean	SD	Median	IQR	*p*-Value ^1^
Day	Starchy foods	7.4	3.3	7.2	4.9–10.0	7.3	3.4	6.5	4.8–9.9	0.633
	Fruit and vegetables	3.0	2.1	2.1	1.6–3.6	3.1	2.8	1.8	1.0–4.5	0.307
	Meat and meat products	6.9	4.8	6.4	3.4–8.8	7.1	4.8	6.0	4.0–10.2	0.414
	Dairy products ^2^	12.0	7.5	9.0	6.4–14.2	11.6	7.3	10.7	7.0–13.3	0.420
	Pastry mainly animal	4.9	4.3	3.5	2.2–5.6	4.7	3.8	3.9	1.9–6.3	0.512
	Protein-energy drink					10.8	9.2	12.7	0.0–19.7	
	Sweet protein cream					5.1	3.4	5.8	1.9–8.9	
	Savory protein cream					2.2	2.8	0.4	0.0–4.3	
Breakfast	Starchy foods	4.0	1.9	3.9	2.4–5.3	3.8	1.8	3.6	2.4–5.0	0.276
	Dairy products ^2^	4.2	4.4	2.3	1.5–5.9	4.5	4.3	2.6	1.5–6.5	0.330
	Protein-energy drink					6.0	5.8	5.5	0.0–10.0	
	Sweet protein cream					0.0	0.0	0.0	0.0–0.0	
	Savory protein cream					0.0	0.0	0.0	0.0–0.0	
Lunch	Starchy foods	1.3	0.9	1.1	0.6–2.1	1.3	0.9	1.1	0.6–1.9	0.640
	Fruit and vegetables	1.4	1.2	1.1	0.5–2.0	1.8	1.6	1.2	0.6–2.5	0.563
	Other mainly plant	0.7	0.8	0.4	0.0–1.2	1.2	1.7	0.5	0.0–2.0	0.149
	Meat and meat products	4.1	3.5	3.7	1.4–6.2	4.1	3.3	3.7	1.6–5.9	0.910
	Dairy products ^2^	0.7	1.3	0.0	0.0–1.2	1.5	1.4	1.1	0.7–2.1	<0.001
	Pastry mainly animal	0.8	1.1	0.4	0.0–1.3	0.5	0.5	0.4	0.1–0.8	0.248
	Other mainly animal	1.3	1.6	0.7	0.1–2.0	1.0	1.4	0.3	0.1–1.3	0.255
	Fish and seafood	1.3	2.0	0.0	0.0–2.0	1.4	1.8	0.6	0.0–2.3	0.328
	Protein-energy drink					1.6	2.6	0.0	0.0–2.5	
	Sweet protein cream					3.6	3.1	3.6	0.3–6.3	
	Savory protein cream					2.2	2.7	0.3	0.0–4.3	
Dinner	Starchy foods	2.1	1.5	2.1	0.9–3.2	2.2	1.9	2.1	0.1–3.7	0.874
	Fruit and vegetables	1.2	1.7	0.4	0.2–1.3	1.1	2.0	0.2	0.1–0.8	0.111
	Meat and meat products	2.4	2.2	1.8	0.0–4.1	2.6	2.5	1.8	0.0–4.5	0.357
	Dairy products ^2^	4.8	3.5	4.2	2.4–6.4	3.8	3.9	3.1	1.6–4.8	0.009
	Pastry mainly animal	2.2	3.4	0.4	0.3–3.7	2.6	3.3	0.9	0.1–4.4	0.519
	Protein-energy drink					1.4	1.8	0.0	0.0–2.3	
	Sweet protein cream					0.3	0.7	0.0	0.0–0.2	
	Savory protein cream					0.1	0.2	0.0	0.0–0.0	
Snacks	Fruit and vegetables	0.3	0.3	0.2	0.0–0.4	0.1	0.2	0.0	0.0–0.1	0.001
	Pastry mainly plant	0.8	0.8	0.7	0.3–1.1	1.4	1.1	1.2	0.6–2.0	0.002
	Other mainly plant	0.3	0.5	0.2	0.1–0.3	0.3	0.5	0.2	0.1–0.3	0.700
	Dairy products ^2^	2.2	2.1	1.7	0.9–2.8	1.8	1.6	1.4	0.8–2.3	0.255
	Pastry mainly animal	1.7	1.2	1.7	0.6–2.3	1.5	1.3	1.2	0.7–1.8	0.499
	Protein-energy drink					1.8	2.7	0.0	0.0–3.0	
	Sweet protein cream					1.2	1.5	0.6	0.0–1.9	
	Savory protein cream					0.0	0.0	0.0	0.0–0.0	

^1^ Wilcoxon-signed rank test. ^2^ intervention modules not included. Only usual food sources that contributed to at least 5% to daily or meal intake are presented. IQR = Interquartile range, SD = Standard deviation.

## Data Availability

The data presented in this study are available on request from the corresponding author. The data are not publicly available due to due to residents or legal representatives not giving full consent.

## Supplementary Materials

The following are available online at https://www.mdpi.com/article/10.3390/nu13072168/s1, Table S1. Intervention levels and their energy and protein content, Table S2: Daily and mealtime protein intake (g) from usual food sources, plant-, animal-based sources, and intervention products during usual care and intervention phase
