# Supplementary material for: Usual Protein Intake Amount and Sources of Nursing Home Residents with (Risk of) Malnutrition and Effects of an Individualized Nutritional Intervention: An enable Study
Source: Nutrients. 2021 Jun 24;13(7):2168. doi: 10.3390/nu13072168 (PMC8308345; doi:10.3390/nu13072168)
Supplement: Supplementary file 1 [file nutrients-13-02168-s001.zip › nutrients-1264144-supplementary.pdf]

nutrients

Supplementary Materials

Table S1. Intervention levels and their energy and protein content

Level	Intervention modules	Energy [kcal]	Protein [g]
1	Sweet protein cream	+125	+10
2	(Sweet **and** savoury protein cream) **or** protein-energy drink	+220-250	+20-22
3	Sweet protein cream **and** protein-energy drink	+345	+32
4	Sweet **and** savoury protein cream **and** protein-energy drink	+470	+42

Protein creams: 40 g per portion, Protein-energy drink: 250 mL per portion.

Table S2. Daily and mealtime protein intake (g) from usual food sources, plant-, animal-based sources and intervention products during usual care and intervention phase

		Usual care phase				Intervention phase				
	Protein intake from	Mean	SD	Median	IQR	Mean	SD	Median	IQR	p-value[1]
Day	Usual food	40.7	10.1	40.4	31.3-48.5	41.8	11.0	41.5	33.0-49.3	0.434
	Usual food (g/kg BW/d)	0.70	0.18	0.70	0.59-0.82	0.72	0.20	0.70	0.59-0.80	0.349
	Plant-based	13.5	4.0	12.7	10.9-16.4	14.4	5.2	14.0	10.2-17.8	0.105
	Animal-based	27.2	8.7	28.4	19.0-32.9	27.4	8.4	26.7	21.4-33.6	0.798
	Intervention					18.2	9.6	17.7	12.2-24.4	
Breakfast	Usual food	9.7	4.8	8.5	6.4-11.7	9.7	4.8	9.2	6.2-11.9	0.944
	Plant-based	4.7	2.0	4.3	3.2-6.1	4.5	1.9	4.4	3.2-6.0	0.410
	Animal-based	5.0	4.4	3.6	1.8-8.4	5.2	4.4	4.3	1.8-7.4	0.472
	Intervention					6.0	5.8	5.5	0.0-10.0	
Lunch	Usual food	12.6	5.6	11.4	8.4-15.6	13.4	5.2	12.5	10.2-16.3	0.346
	Plant-based	4.1	2.0	4.0	2.6-5.3	4.6	2.4	4.3	3.2-5.5	0.237
	Animal-based	8.5	4.9	8.3	5.4-10.8	8.8	4.5	8.7	5.6-10.5	0.707
	Intervention					7.4	4.5	8.0	3.6-10.7	
Dinner	Usual food	13.1	4.7	12.7	11.2-15.8	13.6	5.2	12.7	9.7-15.2	0.591
	Plant-based	3.3	2.0	3.0	1.9-4.5	3.5	2.2	3.2	1.7-5.2	0.767
	Animal-based	9.8	4.1	10.1	6.7-12.4	10.1	4.7	9.4	5.8-12.2	0.667
	Intervention					1.8	1.9	1.4	0.0-3.0	
Snacks	Usual food	5.3	2.3	5.0	4.1-6.0	5.1	2.3	4.8	4.1-5.9	0.667
	Plant-based	1.4	0.9	1.3	0.8-1.7	1.8	1.2	1.7	0.9-2.6	0.025
	Animal-based	3.9	2.4	3.5	2.6-5.1	3.4	2.1	2.9	2.1-4.0	0.080
	Intervention					3.0	3.2	2.5	0.0-4.0	

[1] Total: T-test for paired samples; Plant-/ Animal-based sources: Wilcoxon-signed rank test (n=40).
BW=Body weight, d=Day, IQR=Interquartile range, SD=Standard deviation.
